# Mangosteen (*Garcinia mangostana*) Pericarp and Leaf Tinctures Inhibit LPS-Induced Pro-Inflammatory Responses in Macrophages and Activate Nrf2

**DOI:** 10.3390/nu18030537

**Published:** 2026-02-05

**Authors:** Restituto Tocmo, Mirielle C. Nauman, Yunying Huang, Pradeep Subedi, Jeremy James Johnson

**Affiliations:** Department of Pharmacy Practice, University of Illinois Chicago, Chicago, IL 60612, USA; r.tocmo@reading.ac.uk (R.T.); mirielle.fallon@stjude.org (M.C.N.); 2015687009@gzhmu.edu.cn (Y.H.); subedi@uic.edu (P.S.)

**Keywords:** mangosteen, *Garcinia mangostana*, xanthones, α-mangostin, γ-mangostin, anti-inflammatory, antioxidant

## Abstract

**Background/Objectives**: Xanthones from the tropical fruit mangosteen (*Garcinia mangostana*) have been reported to modulate oxidative stress and inflammatory responses. This work explored the anti-inflammatory potential of mangosteen in the form of tinctures. **Methods**: Tinctures were prepared from the pericarp and leaves, characterized for their major constituents, and evaluated for their in vitro, anti-inflammatory and antioxidant potential. **Results**: HPLC analysis revealed eight major isoprenylated xanthones whose concentrations increased with an increasing alcohol percentage. α-Mangostin and γ-mangostin, two major xanthones present in the tinctures, were stable for 12 weeks at room and elevated (40 °C) temperatures, indicating stability of the tincture. In vitro luciferase reporter assays using HepG2-ARE revealed an alcohol concentration-dependent activation of Nrf2 by pericarp and leaf tinctures. The tinctures inhibited lipopolysaccharide (LPS)-induced production of nitric oxide (NO) and reactive oxygen species (ROS) in RAW264.7 cells. Garcinone C (GarC) and garcinone D (GarD) caused significant inhibition of LPS-induced NO production and iNOS expression. GarC and GarD also induced nuclear translocation of Nrf2 and upregulated heme oxygenase 1 (HO-1), NAD(P)H quinone dehydrogenase 1 (NQO1), and glutathione S-transferase Pi 1 (GSTP1) in RAW264.7 cells. **Conclusions**: Taken together, mangosteen tinctures are a significant source of prenylated xanthones with anti-inflammatory and antioxidant potential.

## 1. Introduction

The mangosteen tree (*Garcinia mangostana*) has been cultivated for centuries in tropical areas of the world, particularly in Southeast Asia. The edible portion (aril) of the fruit comprises only about 25% of the total volume. The remainder of the fruit consists of tough, purple pericarp known to contain a variety of polyphenolic xanthones [1,2]. In addition to the pericarp, other parts of the tree, including the leaves and bark, have been used as folk medicine for hundreds of years [2]. The xanthone-rich pericarp has been traditionally used to treat diarrhea, fevers, dysentery, and abdominal pain, as well as intestinal and skin ailments [3,4]. Mangosteen leaves have been used by Southeast Asian natives as tea to treat diarrhea, dysentery, fever, and thrush. Several studies, including our own, have revealed distinct pharmacological properties of xanthones that include inhibiting cyclin-dependent kinases 2 and 4 [5,6], disrupting the androgen receptor functionality [7], enhancing intestinal barrier function, and modulating immune responses in healthy human subjects [8,9,10,11].

The mangosteen fruit is a popular botanical dietary supplement in the United States and is primarily sold in the form of fruit juice or dietary supplement capsules containing powdered pericarp [12,13]. In selected products, including dietary supplements, the extract may be standardized to xanthones, including α- and γ-mangostin [12]. Based on our own studies as well as those of others, α-mangostin is the most abundant xanthone followed by several other xanthones, including gartanin, 8-deoxygartanin, garcinone C, and garcinone D, among others [14]. The long history and use of mangosteen as a folk medicine, dietary supplement, and juice product suggest it is safe and tolerable [2].

The anti-inflammatory action of mangosteen has been well documented in several in vitro and in vivo studies. Mangosteen extract and two of its major prenylated xanthones, α-mangostin and γ-mangostin, were previously shown to display anti-inflammatory effects by reducing cyclooxygenase-2 (COX-2), pro-inflammatory cytokines (IL-6, IL-1β, IL-4), and nitric oxide (NO) in LPS-induced RAW 264.7 cells [15,16]. Several studies have also demonstrated the anti-inflammatory properties of mangosteen extracts, α-mangostin, and γ-mangostin in animal models (e.g., mice, rats) [10,17,18]. For example, mangosteen extract and α-mangostin were shown to prevent dextran sulfate sodium (DSS)-induced colitis in mice [10,19,20]. These anti-inflammatory properties can be explained through the downregulation of NF-κB and MAPK signaling pathways, as well as induction of the antioxidant response as demonstrated in immortalized cell lines (i.e., RAW264.7, IEC-6) and mouse colitis mode [10,17,19].

Tinctures or alcoholic extracts of plants have a long history dating back to the ancient Egyptians. They are administered as ingredients of many liquid formulations. The European Pharmacopoeia contains a monograph about tinctures in which their modes of preparation and the amounts of the herbal drugs in the tinctures are specified [21]. In the herbal drug monographs of the European Pharmacopoeia, the amount of alcohol required to facilitate extraction of the phytochemical is not specified, and thus herbal drug tinctures contain a range of concentrations of alcohol (20 to 60%, *v*/*v*) [22]. Such variability in alcohol content can influence the efficiency of phytochemical extraction. Accordingly, the present study employed a tincture-based extraction using aqueous ethanol of varying concentrations, providing a practical and phytochemically rational approach for obtaining bioactive extracts suitable for subsequent biological screening and functional evaluation.

There are examples of mangosteen available as an alcoholic tincture in the commercial market with limited evaluation in the scientific literature. In this work, we prepared tinctures from the pericarp and leaves of mangosteen obtained from the Philippines. Our goals were to assess the qualitative and quantitative profiles of the constituents of the tinctures, identify their active constituents, and investigate their potential anti-inflammatory mechanism via inhibition of pro-inflammatory responses and activation of the Nrf2 pathway in macrophages. In addition, we evaluated the stability of two major constituents of the tinctures.

## 2. Materials and Methods

### 2.1. Chemicals

Dulbecco’s Modified Eagle Medium (DMEM; with L-glutamine) (Thermo Fisher Scientific, Chicago, IL, USA), trypsin–ethylenediaminetetraacetic acid (EDTA) and penicillin–streptomycin solutions were purchased from Life Technologies Co. (Grand Island, NY, USA); RIPA (10×) lysis buffers were purchased from Cell Signaling Technology (CST, Danvers, MA, USA); 2′,7′-dichlorofluorescin diacetate (Cas. 4091-99-0) was purchased from Sigma-Aldrich (St. Louis, MO, USA); fetal bovine serum (FBS) was obtained from Atlanta Biologicals Inc. (Lawrenceville, GA, USA); and Prolong Gold Antifade with DAPI (Cat # 8961S) was purchased from CST. All other chemicals and solvents were of analytical grade and were purchased either from Sigma-Aldrich (St. Louis, MO, USA) or Thermo Fisher Scientific (Chicago, IL, USA).

### 2.2. Plant Collection

*Garcinia mangostana* plant samples were provided by Dr. Alfred Villarico. These materials were collected in a mangosteen plantation located in the city of Kidapawan, Province of Cotabato, Southern Philippines in October of 2019. Plant parts were divided into fruit pericarp and aerial organs (stem bark and leaves), oven dried at 50 °C until complete dryness, milled, and stored at −20 °C until use.

### 2.3. Solvent Extraction

Methanolic extracts were obtained by extractions of 0.5 g dried samples with HPLC-grade methanol (MeOH, 1:10 sample to solvent ratio, *w*/*v*) at room temperature. After centrifugation, the methanolic layer was collected and stored at −20 °C, and extraction was repeated two more times. The organic layers were pooled, further diluted with MeOH, filtered through 0.2 µm PVDF syringe filters, and injected into HPLC for analysis of xanthones. The remaining extracts were kept at −20 °C until further use. Extractions were carried out in triplicate.

### 2.4. Preparation of Tinctures

Tinctures were prepared from dried mangosteen pericarp and leaves ground into powders. Briefly, 100 g of samples in 1 L glass jars were added with 600 mL (1:6 *w*/*v*) of 40, 60, or 80% ethanol (200 proof EtOH in water), mixed vigorously, and stored in the dark for 10 days. The jars were stirred every day. Two batches of tinctures (*n* = 2) for each plant part were prepared. Tinctures (abbreviated as E40, E60, E80) were collected on Day 10 and filtered (Whatman no. 4), transferred into 40 mL amber bottles, and stored at 4 °C until further use.

### 2.5. HPLC Analysis

Solvent extracts from pericarp and leaves, as well as the tinctures, were analyzed for their xanthone composition by HPLC. Methanolic extracts and tinctures were diluted with appropriate volumes of MeOH, filtered through 0.2 µm PVDF syringe filters and injected into HPLC, following our previously described HPLC conditions [9] (Tocmo et al. 2021). An Ultra-Fast Liquid Chromatograph (UFLC, Shimadzu, Columbia, MD, USA) system equipped with a photodiode array detector and a Phenomenex Kinetex C18 column (250 mm × 4.60 mm i.d., µm; Torrance, CA, USA). To quantify xanthones in the samples, highly pure xanthones (>90%) previously isolated by semi-preparative chromatography [9] (Tocmo et al. 2021) were used to create standard calibrations. Standard curves (R^2^ = 0.99) of eight xanthones were prepared using the same HPLC conditions as the samples.

### 2.6. Evaluation of Metabolic Activity by MTT

Fifteen mL of tinctures (about 1 tablespoon) were dried using a rotary evaporator in vacuo (35 °C) and subsequently lyophilized in a Lyovapor™ L-200 (Buchi, New Castle, DE, USA) freeze dryer. Stock solutions of dried extracts dissolved in dimethyl sulfoxide (DMSO) were prepared and further diluted in DMEM (0.1% DMSO final concentration). The metabolic activity of solvent extracts and tinctures were assessed in murine macrophage (RAW264.7) and human hepatocellular carcinoma (HepG2-ARE) cell lines (ATCC). Cells were maintained in DMEM (Gibco, Waltham, MA, USA) following our previously described cell culture conditions [23]. Briefly, cells were seeded in 96-well plates at an initial density of 1 × 10^4^ cells/well and 2 × 10^4^ cells/well, respectively. After 24 h, cells were treated with the extracts (0–500 µg/mL) and cell viability was measured by MTT (3-(4,5 dimethylthiazol-2-yl)-2,5-diphenyltetrazolium bromide) assay following our previously reported protocol [23]. DMSO (0.1% in DMEM) was used as the negative control. Percent viability results were expressed in terms of cell viability (%) relative to the controls, and the toxicity profile was described as IC_50_ values.

### 2.7. Inhibition of NO Production

The concentration of nitrite (NO_2_^−^), an indicator of NO production, was measured using the standard Griess assay. Cells were treated with 0–20 μg/mL of tinctures or 0–20 μM of compounds for 24 h with or without 0.2 μg/mL LPS and washed with PBS prior to adding Griess reagent (reagent A: 1% sulfanilamide in 5% phosphoric acid; reagent B: 0.1% N-1-napthylethylene-diamine dihydrochloride in water; 1/1, *v*/*v*). The absorbance at 545 nm was determined after 10 min incubation in a micro-plate reader (Molecular Devices, Sunnyvale, CA, USA). NO production was expressed as % of the untreated sample (control).

### 2.8. ARE Activation Assay

Nrf2 antioxidant response was measured using a recombinant HepG2-ARE with stably integrated luciferase firefly gene under the control of ARE promoters. Briefly, cells (2 × 10^4^ cells/well) were seeded into two 96-well plates (flat clear bottom white polystyrene, Corning, Corning, NY, USA) for 24 h (80% confluence); one plate was used to determine ARE activation and the other to measure cell viability following a standard MTT assay. Cells were then exposed to titrated or fixed concentrations of mangosteen tincture extracts (0–75 μg mL^−1^) dissolved in DMSO (0.1% final concentration) for 24 h. For assessment of Nrf2 activation, luciferase assay reagent (Bright-Glo Luciferase Assay) (Promega, Chicago, IL, USA) was directly added to the well containing treatment medium at 1:1 ratio (100 μL per well containing 100 μL treatment media). The Bright-Glo reagent causes cell lysis and generates a luminescent signal, which is proportional to ARE activation. Total luminescence was measured after 2 min using a luminometer (BD moonlight 3010, BD Biosciences, Durham, NC, USA). Nrf2 activation (expressed in relative luminescence units, RLU) was calculated as the fold increase in luminescence relative to the DMSO control after having normalized to cell viability.

### 2.9. Western Blot

Protein concentration was measured with a BCA protein assay kit (R&D Systems, Minneapolis, MN, USA) according to the manufacturer’s instructions. After separation by SDS-PAGE, proteins were transferred onto a 0.2 μm nitrocellulose membrane. Western blot analysis was performed as previously described [9]. Blocked membranes were incubated with iNOS (18985-1-AP), Nrf2 (16396-1-AP), heme oxygenase 1 (HO-1; 10701-1-AP), glutathione *S*-transferase Pi 1 (GSTP1, #3369), NAD(P)H quinone dehydrogenase 1 (NQO1; 11451-1-AP) and β-actin antibodies purchased from ProteinTech (Rosemont, IL, USA) or Cell Signaling Technology (Danvers, MA, USA). Membranes were incubated overnight at 4 °C followed by a 1 h incubation with anti-rabbit IgG, horseradish peroxidase (HRP)-conjugated secondary antibody (#7074, CST) or mouse IgGκ binding protein-HRP (#7076, CST). Blot images were captured using a FluorChem E imager (ProteinSimple, San Jose, CA, USA). The intensities were quantified by densitometric analysis using AlphaView software (v4.1.4) for FluorChem™ systems.

### 2.10. Stability Study

The pericarp tincture (80% pericarp) was selected for stability study. Tinctures (30 mL) were poured into amber glass containers and stored either in the refrigerator (4 °C), on a shelf at room temperature (~23 °C) covered with aluminum foil, or in a shaking incubator at 40 °C. Tinctures were first analyzed by HPLC fresh after preparation (Day 0), and samples (*n* = 2) from each temperature treatment were obtained every 7 days for 3 months (12 weeks) for HPLC analysis of the two most abundant xanthones, α- and γ-mangostin.

### 2.11. Statistical Analysis

Statistical analyses were performed using SigmaPlot 13 software (Systat Software, Inc., San Jose, CA, USA). Data from at least three independent experiments were subjected to one-way ANOVA followed by a post hoc analysis with Tukey’s test to determine significant differences among treatments. Differences at *p* < 0.05 were considered statistically significant. Results are presented as mean values ± SD.

## 3. Results

### 3.1. Identification and Quantification of Xanthones

**Figure 1:**
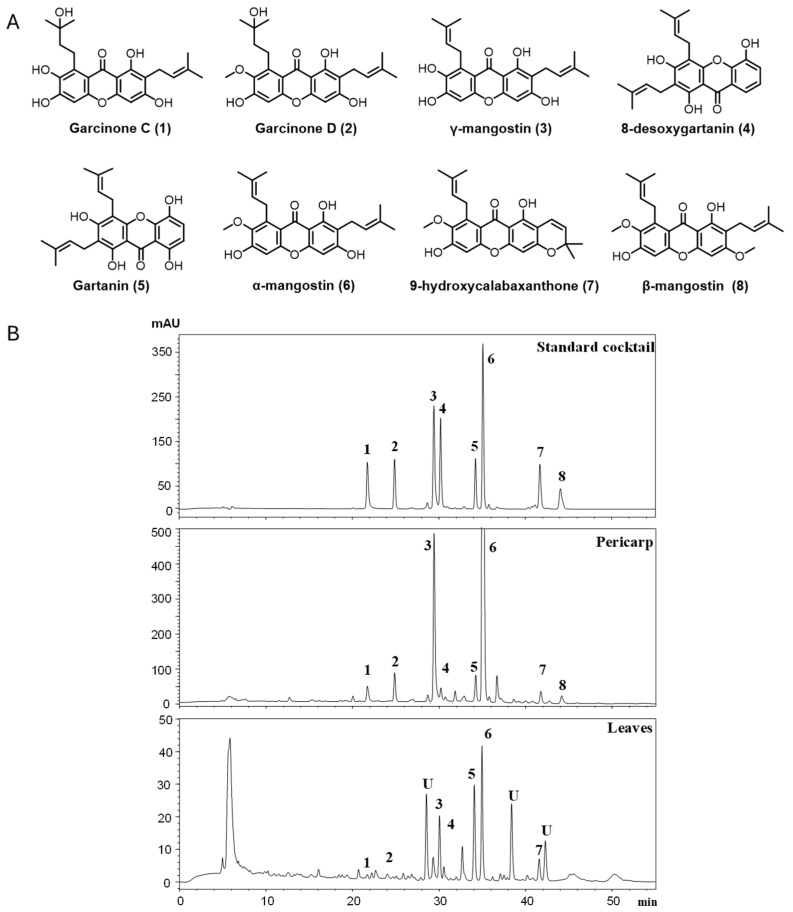
Chemical structures of xanthones from *Garcinia mangostana* (**A**) and representative chromatograms of xanthones from methanolic extracts of mangosteen fruit pericarp and leaves (**B**). Number assigned to each peak corresponds to the compound listed in Table 1.

To determine the chemical constituents of mangosteen pericarp and leaves, methanolic extracts were prepared and analyzed by HPLC (Figure 1). Extracts were spiked with pure xanthones (previously isolated and characterized [9]) and analyzed using the same HPLC conditions as the unspiked samples. Comparison of peak retention times revealed eight prenylated xanthones in the methanolic extracts and were identified as garcinone C (1), garcinone D (2), γ-mangostin (3), 8-deoxygartanin (4), gartanin (5), α-mangostin (6), 9-hydroxycalabaxanthone (9-OHCal, 7), and β-mangostin (8). α-Mangostin (58 ± 3.8 mg/g, db) and γ-mangostin (8 ± 2.3 mg/g, db) were the most abundant compounds and were found to be 67 and 26 times higher in the pericarp than in the leaves (Table 1). The other six compounds were all significantly lower in the leaf extracts than in the pericarp. Three peaks in leaf extracts (labeled “U” in Figure 1B) were not identified due to their relatively low concentrations.

**Figure 2:**
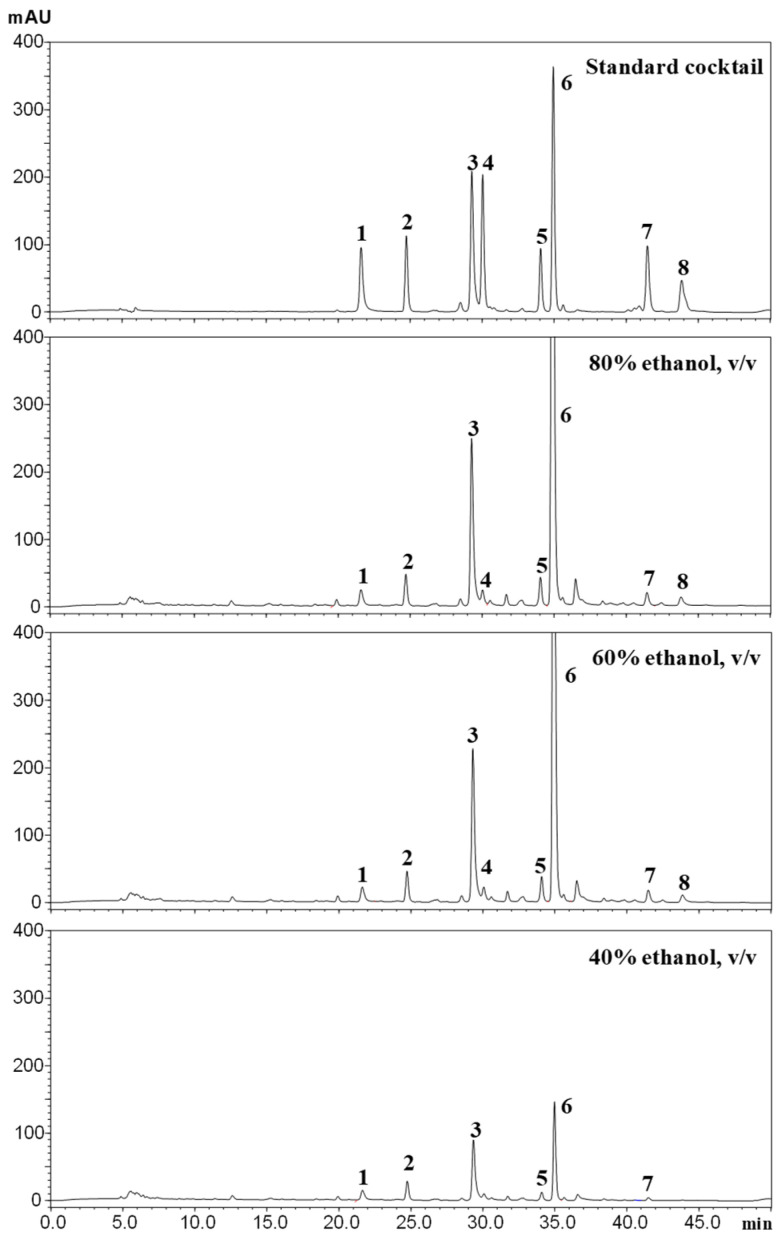
Representative chromatograms of xanthones from the pericarp prepared using ethanol concentrations of 40%, 60%, and 80% (*v*/*v*). Number assigned to each peak corresponds to the compound listed in Table 1.

Based on the presence of prenylated xanthones in the methanolic extracts of the pericarp and leaves, tinctures were prepared according to the procedures recommended by the EU Pharmacopoeia. The procedure involved extraction of 1 part dried *G. mangostana* with 6 parts of alcohol aqueous solutions (200 proof) at alcohol concentrations of 40, 60, and 80% *v*/*v*. HPLC analysis of the pericarp tinctures (Figure 2) revealed the same eight major peaks identified in the methanolic extracts (Figure 1). Concentrations of α-mangostin and γ-mangostin were 26 and 3 mg/g in E80P (Table 2) compared with 58 and 8 mg/g in the methanolic extract (Table 1). The concentrations of the other six compounds were significantly lower in all tincture preparations compared with the methanolic extracts. HPLC quantification revealed alcohol concentration dependence of xanthone levels in the tinctures, with both E80P and E80L containing significantly higher levels compared with E60 and E40. It is notable that while there is a statistical difference (*p* < 0.05) between E80P and E60P, the concentrations of all eight xanthones between them differed only by a range of 9 to 44%. There was, however, a marked decrease in the concentrations of all eight compounds in E40 tinctures for both pericarp and leaves. Based on these results, 40% EtOH aqueous solution does not appear suitable for the preparation of xanthone-rich tinctures from mangosteen pericarp and leaves.

### 3.2. Thermal Stability of Tinctures Under Normal and Accelerated Conditions

**Figure 3:**
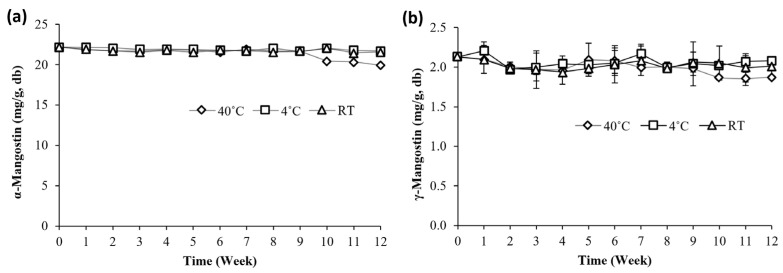
Stability of (**a**) α- and (**b**) γ-mangostin in 80% pericarp tincture over a 12-week period at 4 °C, room temperature (RT), and 40 °C.

α-Mangostin and γ-mangostin, two of most abundant xanthones in the tinctures, were selected for HPLC monitoring of thermal stability over a 3-month period. For tinctures stored at 4 °C and room temperature (RT), concentrations of α-mangostin at Week 12 (W12) were 21.8 mg/g and 21.6 mg/g, respectively, compared with 22.3 mg/g at Day 0 (D0) (Figure 3a). There was an indication of a slight downward trend in α-mangostin concentrations at W10 (20.42 mg/g) to W12 (19.93 mg/g). Concentrations of α-mangostin at W12 corresponded to a 90% retention relative to D0. A similar trend was observed for γ-mangostin whose concentrations remained the same at D12 for both 4 °C (2.1 mg/g) and RT (2.01 mg/g) compared with D0 (2.13 mg/g) (Figure 3b). At 40 °C, a decrease in the levels of γ-mangostin was observed at W9 (1.98 mg/g) to W12 (1.87 mg/g) that corresponds to a 12.2% reduction in concentration relative to D0. These data suggest that, while γ-mangostin levels remained high after 3 months under 40 °C, it showed indications of a slightly higher sensitivity to elevated temperatures than α-mangostin at the same storage temperature. There were no new peaks observable during the duration of the study suggesting that no new compounds were formed.

### 3.3. Activation of the Antioxidant Response Element (ARE) by Mangosteen Tinctures

**Figure 4:**
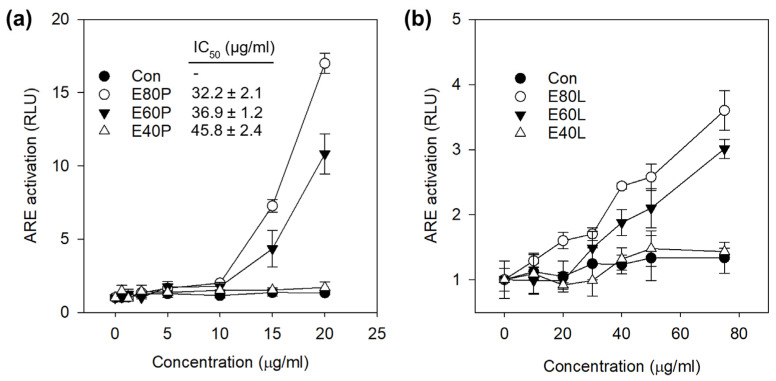
Effect of alcohol tinctures (40, 60, and 80% alcohol) on the activation of antioxidant response element (ARE) in HepG2-ARE cells by tinctures from the mangosteen (**a**) pericarp and (**b**) leaves.

The ability of the tinctures to activate the Nrf2 pathway was evaluated using a luciferase reporter assay. To determine a non-cytotoxic concentration range, ARE-luciferase reporter cells (HepG2-ARE) were exposed to 0–100 μg/mL of tinctures followed by a standard MTT assay. Pericarp tinctures exhibited alcohol concentration- and dose-dependent metabolic activity with IC_50_ values of 35 µg/mL (E80P), 37 µg/mL (E60P), and 46 µg/mL (E40P) (Figure 4). All leaf tinctures were non-cytotoxic up to 100 µg/mL. Based on these results, 20 µg/mL and 100 µg/mL were selected as the highest treatment concentrations for the luciferase assay. The concentration to quadruple (CQ) the induction of the luciferase gene was 11.2 and 12.8 µg/mL for E80P and E60P, respectively. E40P and E80/60/40L did not reach a CQ at the concentrations tested but exhibited moderate Nrf2 activation (1.4 to 3.6 folds) relative to the control.

### 3.4. Inhibition of Pro-Inflammatory Markers in LPS-Activated Macrophages

**Figure 5:**
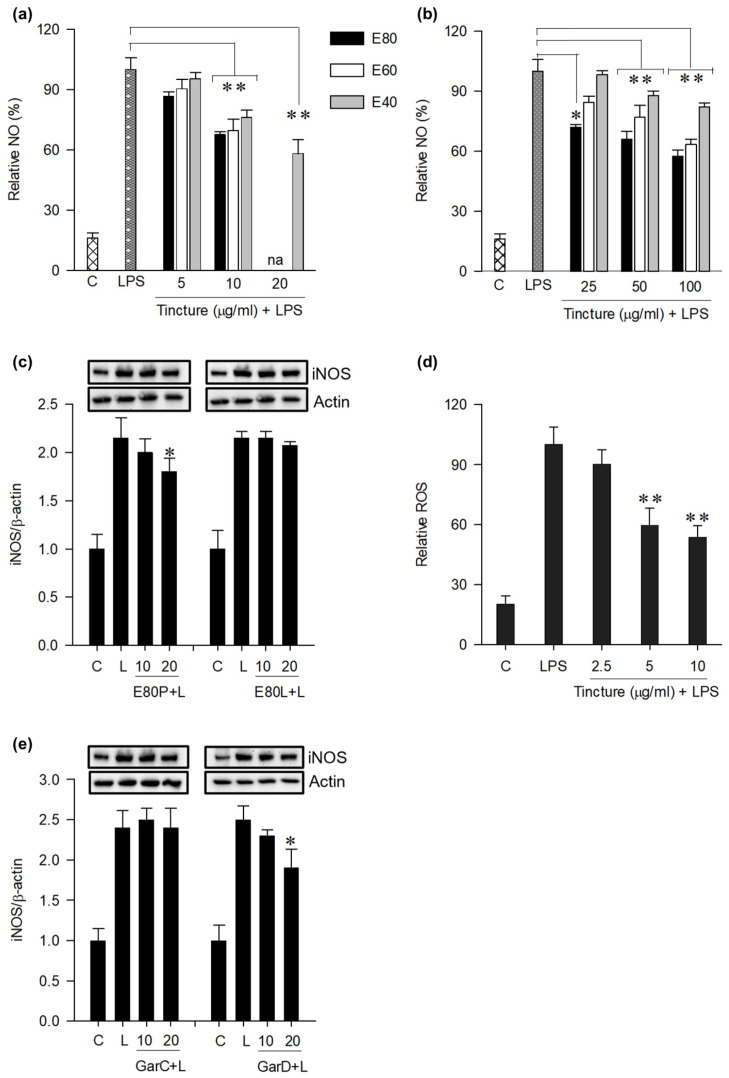
Alcohol tinctures from mangosteen (**a**,**b**) pericarp and leaves dose-dependently inhibit LPS-induced NO generation in RAW 264.7 cells. (**c**) iNOS. (**d**) Inhibition of ROS in LPS-activated RAW cells by 80% pericarp tincture (E80P). (**e**) Effect of GarC and GarD on LPS-induced iNOS production. Data are expressed as mean values ± SD from triplicate experiments. Bars bearing * (*p* ≤ 0.05) and ** (*p* ≤ 0.01) were significantly different by one-way ANOVA followed by Tukey’s HSD test vs. LPS.

NO and inducible nitric oxide synthase (iNOS) are markers of inflammatory response [24]. To test the anti-inflammatory effect of the tinctures, NO and iNOS were measured in LPS-activated RAW cells in the presence and absence of tinctures. Exposure of RAW cells to 200 ng/mL LPS (24 h) induced a 5-fold increase in NO levels in the culture medium. Pre-treatment with tinctures (0–20 μg/mL) exhibited an alcohol concentration- and dose-dependent inhibition of NO (Figure 5a). At 10 μg/mxl, E80P and E60P inhibited NO by 30% relative to LPS-treated cells. At 20 μg/mL, E80P and E60P were cytotoxic (IC_50_ = 19.35 μg/mL) against RAW cells and were, therefore, not tested. E40P inhibited NO production by 40% relative to LPS treatment. Similar trends were observed with leaf tinctures (Figure 5b), albeit NO inhibition was achieved at a significantly higher non-cytotoxic concentration range (25–100 μg/mL) compared with the pericarp tinctures. At 100 μg/mL, E80L and E60L decreased NO by 40% relative to LPS treatment. Based on NO inhibition data, E80P and E80L were selected to test whether the tinctures inhibit iNOS in LPS-activated RAW cells. Western blotting revealed that LPS-stimulated RAW cells had iNOS levels two-fold higher than the untreated control (Figure 5c). E80P at 10 μg/mL caused a 16% decrease in iNOS levels relative to LPS treatment. E80L did not decrease iNOS levels, suggesting that the NO inhibition by the tinctures (Figure 5a,b) was not completely attributable to the inhibition of iNOS by the tinctures.

Many chronic inflammatory diseases are related to high levels of ROS [25]. To further evaluate the anti-inflammatory potential of mangosteen tinctures, the effect of E80P on ROS production in LPS-activated macrophages was quantified using the oxidation-activated fluorescent dye DCFH-DA. LPS treatment induced a 5-fold increase in ROS relative to the control (Figure 5d). Treatment of RAW cells with E80P (0–20 μg/mL, 24 h) caused a dose-dependent decrease in DCF fluorescence, reaching a 46% reduction (vs. LPS) at 10 μg/mL, indicating an inhibitory effect of tinctures against LPS-induced ROS production.

### 3.5. Garcinone D and Garcinone C Inhibit NO in LPS-Activated Macrophages

**Figure 6:**
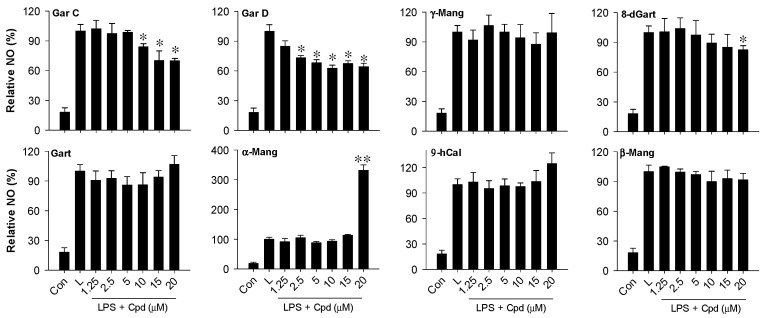
Effect of individual xanthones on LPS-induced NO generation in RAW 264.7 cells. * *p* < 0.05, ** *p* < 0.001.

To identify the potentially active anti-inflammatory compound(s) in the tinctures, eight major xanthones (Figure 1) were tested for their ability to inhibit NO in LPS-activated RAW cells. We have previously isolated and characterized all these xanthones by LC-MS and NMR and reported on their antioxidant mechanism in HT-29 cells [9]. Treatment of RAW cells with non-cytotoxic levels (0–20 μM) of individual compounds revealed that only three (GarC, GarD, and 8-deoxygartanin) of eight xanthones displayed moderate inhibitory effects against NO (Figure 6). GarC and GarD had comparable effects, inhibiting NO (vs. LPS, 20 μM) by 35 and 38%, respectively. At 20 μM, 8-deoxygartanin only caused a 12% reduction in NO levels compared with the LPS treatment. Interestingly, α-mangostin did not inhibit NO at 1.25 to 15 μM but caused a 3-fold increase in NO levels (vs. LPS) at 20 μM. Gartanin and 9-OHCal also exhibited a moderate but not significant increase in NO levels at 20 μM compared with the untreated controls.

### 3.6. Garcinone D and Garcinone C Activate the Nrf2 Pathway in Macrophages

**Figure 7:**
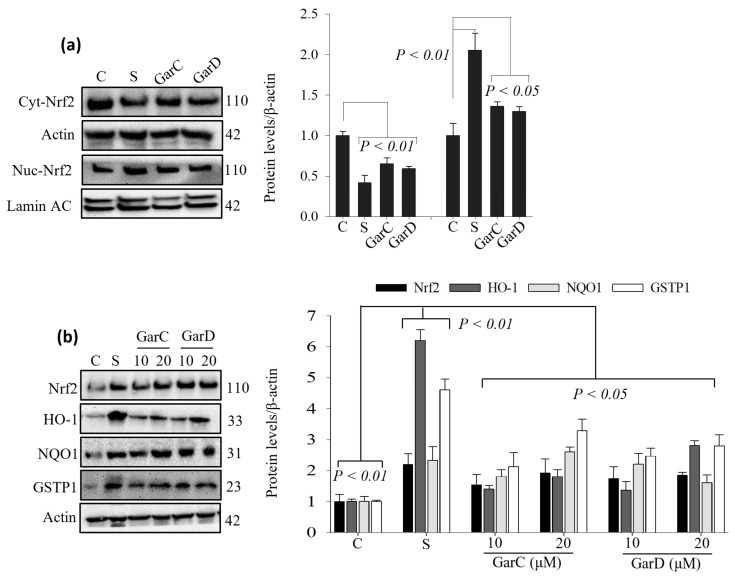
(**a**,**b**) Garcinone C and D activate Nrf2 and upregulate the expression of Nrf2-mediated antioxidant enzymes in RAW 264.7 cells. Cells were treated with 10 and 20 µM GarC and GarD over a 24 h period. Cell lysates were collected and immunoblotted with antibodies; relative protein densities normalized to β-actin. Data are expressed as mean values ± SD from triplicate experiments.

The crosstalk between the Nrf2 and NF-κB pathways is a key mechanism that regulates cellular response to oxidative stress and inflammation [25]. We have previously shown that GarD is the most potent ARE activator out of seven isoprenylated xanthones isolated from mangosteen pericarp [9]. Based on this result and our current data on NO inhibition by GarD and GarC, we selected both compounds to evaluate their ability to activate the Nrf2 pathway in macrophages. Sulforaphane (SFN, positive control), a potent Nrf2 activator [26], caused a 58% decrease in cytosolic Nrf2 levels and a 2-fold increase in nuclear Nrf2 levels relative to the controls (Figure 7a). Exposure to 20 µM of GarC and GarD for 3 h decreased cyt-Nrf2 levels by 35% and 41% (vs. controls). Accumulation of Nrf2 in the nucleus was also evident with GarC and GarD increasing nuc-Nrf2 levels (vs. control) by 36% and 30%. Furthermore, GarC and GarD dose-dependently increased the levels of Nrf2 in whole cell lysates and upregulated the levels of Nrf2-mediated antioxidant enzymes HO-1, GSTP1, and NQO1 (Figure 7b).

## 4. Discussion

The present work reports on the qualitative and quantitative chemical profiles, bioactivity, and storage stability of tinctures of mangosteen fruit pericarp and leaves. Eight major prenylated xanthones were identified and quantified, and their in vitro, anti-inflammatory, and antioxidant potentials were evaluated.

HPLC analysis revealed qualitatively similar chemical profiles in the methanolic extracts of mangosteen leaves and pericarp (Figure 1), although the leaf extracts contained three additional unknown peaks that were either undetectable or present in trace amounts in the pericarp. The major peaks were identified as prenylated xanthones—all of which were significantly higher in the pericarp than in the leaves. Our results were in agreement with previous studies identifying all eight xanthones (Table 1 and Table 2) as the commonly detected xanthones in mangosteen pericarp [9,27,28,29]. The chemical profile of mangosteen leaf extracts is not well characterized, and there are only a few studies reporting their antimicrobial [30], melanogenic [31], and anticancer properties [28]. To our knowledge only one paper reports on the xanthone constituents of mangosteen leaves [32]. Ref. [33] identified gartanin (also identified in the present study) and two other xanthones (1,5,8-trihydroxy-3-methoxy-2[3-methyl-2-butenyl] xanthone and 1,6-dihydroxy-3-methoxy-2[3-methyl-2-butenyl] xanthone).

Based on the profiles of the methanolic extracts of leaves and pericarp, tinctures of different alcohol concentrations (40, 60, 80%, *v*/*v*) were prepared and the differences in their chemical constituents were evaluated. HPLC analysis revealed a chemical profile dominated by prenylated xanthones for both leaf and pericarp tinctures similar to those identified in the methanolic extracts (Figure 1 and Figure 2). Quantification using previously isolated xanthones as standards revealed increasingly higher amounts of all xanthones detected as the ethanol ratio increased (Table 2). It can also be noted that the concentrations of all xanthones were significantly higher in pericarp tinctures than in the leaf tinctures. Although there is currently no commercially available mangosteen tincture, our HPLC data will be useful for determining dosage and quality if this form of mangosteen-derived herbal product is developed in the future. Furthermore, the observed stability of α- and γ-mangostin over the 12-week study (Figure 3) indicates stability of the tinctures at room temperature storage.

Mangosteen and its isoprenylated xanthones have received considerable research attention in the context of elevating cellular defenses to oxidative and inflammatory stresses in cells and in vivo. For example, mangosteen pericarp extracts and two major xanthones, α- and γ-mangostin, have all been shown to enhance antioxidant and anti-inflammatory defenses in various cell lines and animal models [15,16,18,34,35,36,37,38]. In many of these studies, ROS scavenging, downregulation of pro-inflammatory markers (NO, cytokines), and upregulation of antioxidant enzymes are typical markers being investigated to indicate downstream biological effects. In terms of the anti-inflammatory mechanisms, common pathways implicated are the NF-κB and MAPK pathways [19,35,39]. The literature in this area is currently dominated by those investigating anti-inflammatory effects of α- and γ-mangostin, as these two xanthones are commercially available. However, our previous study revealed that other isoprenylated xanthones in mangosteen, such as GarD and gartanin, were potent activators of the Nrf2 pathway in the human colorectal adenocarcinoma cell line (HT-29) [9]. Although studies have shown anti-inflammatory effects of mangosteen extracts and xanthones in macrophages [16,18,39], there is little information on whether mangosteen extracts and its major xanthone constituents could activate Nrf2 in macrophages. Our findings support our hypothesis that xanthone-rich tinctures can enhance antioxidant defense in LPS-activated RAW264.7 cells through the activation of Nrf2.

We routinely used non-cytotoxic levels of tinctures (0–20 μg/mL) and xanthones (0–20 μM) to elicit the observed anti-inflammatory responses in RAW cells. Pre-treatment of cells with tinctures prior to LPS (0.2 μg/mL) activation attenuated the production of inflammatory markers, including NO, iNOS (Figure 5a–c), and ROS (Figure 5d). In addition, pre-treatment of RAW cells with individual xanthones revealed that only GarC and GarD significantly inhibited LPS-induced NO production (Figure 6). Western blot analysis further revealed that GarD and GarC could moderately inhibit LPS-induced iNOS in RAW cells (Figure 5e). These results support previous findings [18,34,35,39], showing anti-inflammatory effects of mangosteen in murine macrophages. However, it can be noted that α-mangostin at 20 μM further increased NO levels (Figure 6) while the rest of the xanthones did not exhibit inhibitory effects. Taken together, these results suggest that GarC and GarD could be the major anti-inflammatory compounds in the tinctures, whereas α-mangostin may have a pro-inflammatory effect at relatively higher concentrations. These results further highlight the differential effects of structurally similar xanthones present in mangosteen and the importance of the dose or concentration used in in vitro bioactivity determination. GarC and GarD contain four hydroxyl (OH) groups compared with three in α-mangostin. The position and availability of OH in phenolic compounds stabilize their free radical scavenging activity since OH groups are electron donors [40]. In addition, one of the prenyl groups in GarC or GarD is hydroxylated. These differences in chemical structures may explain the observed differences in in vitro anti-inflammatory properties between GarC/D and α-mangostin. These findings reinforce the broader concept that minor structural features within the xanthone scaffold can shift biological outcomes, underscoring the necessity of considering fine structural nuances when interpreting anti-inflammatory potential and predicting functional behavior in complex phytochemical mixtures. We currently do not have experimental data to explain this pro-inflammatory tendency of α-mangostin; however, possible dose-dependent anti- and pro-inflammatory effects of mangosteen xanthones, in general, especially at high concentrations, can be explored in future in vitro and in vivo studies. In this study, our approach to evaluating the biological properties of mangosteen is to deconstruct the plant to individual xanthones to characterize their anti-inflammatory properties. In the future, we will reconstruct the plant by systemically preparing xanthone combinations to characterize the anti-inflammatory properties. It may be possible that the anti-inflammatory xanthones will work in concert with each other to overcome the pro-inflammatory properties that we described.

Several studies in the past decade have demonstrated the inhibition of pro-inflammatory markers (i.e., cytokines, NF-κB) by mangosteen extracts. Therefore, we focused our attention on the potential induction of the Nrf2-mediated antioxidant defense mechanism in RAW cells. Using the ARE-luciferase reporter cell line HepG2-ARE, a dose-dependent increase in luciferase signal caused by both pericarp and leaf extracts was observed (Figure 4a,b). This result suggests that constituents in the tinctures could activate the ARE, a cis-acting enhancer sequence that mediates transcriptional activation of genes in cells exposed to Nrf2 activators and oxidative stress [41,42]. We have previously demonstrated that mangosteen-derived xanthones could differentially activate the ARE and that GarD was the most potent Nrf2 activator of seven tested xanthones [9]. Based on this previous result and our current data revealing NO inhibitory effects of both GarC and GarD (Figure 5 and Figure 6), we selected these compounds and carried out Western blot analysis of Nrf2 and its downstream antioxidant enzymes. Our results revealed that both GarC and GarD induce moderate nuclear translocation of Nrf2 in RAW cells (Figure 7a), indicating activation of the Nrf2 pathway. This finding is consistent with that of a previous study reporting Nrf2 activation in C17.2 neural stem cells by GarD [43]. At present, there are not any reports on GarC activating Nrf2; however, the structural similarity it shares with GarD suggests that GarC may also be an Nrf2 activator. Additional Western blot experiments revealed further evidence of Nrf2 pathway activation through the enhancement of Nrf2 expression itself and upregulation of Nrf2-mediated antioxidant enzymes, including HO-1, GSTP1, and NQO1. LPS-induced activation of the TLR4–NF-κB signaling axis has long been observed to involve the generation of ROS [44,45], as we witnessed (Figure 5d). The activation of Nrf2-mediated enzymes by GarC and GarD can explain the inhibition of LPS-induced ROS (Figure 5d) and NO production (Figure 5a–c and Figure 6), likely through the crosstalk between the NF-κB and Nrf2 pathways [25]. Overall, data presented herein provide evidence that the anti-inflammatory effect of tinctures in RAW cells may partially be attributed to the enhancement of the cellular antioxidant system, a downstream effect of Nrf2 activation. Our results warrant further investigation on the possible interaction between NF-κB and Nrf2 as triggered by mangosteen xanthones to further strengthen evidence on the anti-inflammatory effects of mangosteen tinctures.

Previous studies on xanthone extraction from various plant matrices have established that higher percentages of ethanol in aqueous solvents yield higher yields of these medium-polarity compounds [46,47,48]. Our results demonstrated that the tincture containing the highest ethanol concentration (80%) yielded the most xanthone-rich and bioactive extract, aligning with and confirming this principle. The choice of tincture preparation method using maceration with aqueous ethanol was therefore deliberate and phytochemically rational, targeting efficient xanthone solubility and producing extracts suitable for subsequent biological screening and functional evaluation. However, this approach has certain limitations, including limited selectivity for individual compounds and potential variability in extract composition. The relevance of this optimized approach becomes clear when contrasted with advanced extraction technologies. While methods such as Pressurized Liquid Extraction (PLE) and Supercritical Fluid Extraction (SFE) offer superior throughput for industrial isolation, they require high capital investment and technical expertise [49,50]. Furthermore, the high operational temperatures of PLE may risk degrading heat-sensitive xanthone analogues [51]. The inherently non-polar nature of pure SFE-CO_2_ makes it inefficient for these compounds without polar modifiers, such as ethanol [50,52], effectively incorporating the core solvent of our study into a far more complex system. In contrast, this optimized gradient ethanol tincture offered a direct, economical, and practical method for producing a bioactive xanthone-rich extract, demonstrating that fine-tuning a traditional protocol can yield scientifically validated, application-ready results. Future studies may explore mechanistic comparisons across alternative extraction methods, allowing a more comprehensive evaluation of how different approaches influence extract composition, compound selectivity, bioactivity, and extraction efficiency.

## 5. Conclusions

In conclusion, we propose a scenario involving the activation of Nrf2 and upregulation of its downstream antioxidant proteins, as well as inhibition of pro-inflammatory markers (i.e., NO, iNOS, ROS), to account for the anti-inflammatory mechanisms of mangosteen tinctures in macrophages. Despite the indication that a major xanthone in the tincture (α-mangostin) appears to exacerbate the inflammatory response to LPS, the overall decrease in NO and ROS levels (Figure 5) indicates a net anti-inflammatory effect of the tinctures. This observation points to a potential synergistic effect of the constituents in the tinctures and therefore warrants further investigation into said synergism. Overall, we provide preliminary evidence suggesting possible modulatory effects of mangosteen xanthones on the Nrf2–NF-κB axis. Further cellular experiments involving gene knockdown (i.e., Nrf2, NF-κB), as well as in vivo studies, will clarify the underlying mechanisms of this potential crosstalk. There are additional findings in this study that warrant continued inquiry. The differences in the extent to which individual xanthones inhibit or promote LPS-induced NO production indicate the structure-dependent anti-inflammatory activity of these compounds. The subtle differences in their functional groups (i.e., prenyl, hydroxyl, methoxy groups) may explain said differences in bioactivity; however, detailed qualitative and quantitative structure–activity relationship analyses were beyond the scope of the present study and could be explored in future studies along with comparisons across alternative extraction methods. In addition, our 12-week storage study provides a good indication of the stability of the tinctures, but it is not able to predict shelf life. A long-term (>6 months) storage study would give a better approximation of the shelf life of the tinctures.

## Figures and Tables

**Table 1:** Concentrations of xanthones in the pericarp and leaves of mangosteen.

Peak No.	Retention Time	Compound	Concentration (mg/g, db *)	
			Pericarp	Leaves
1	21.7	Garcinone C	1.32 ± 0.25 ^a^	0.03 ± 0.01 ^b^
2	24.8	Garcinone D	1.57 ± 0.43	tr
3	29.4	γ-mangostin	8.03 ± 2.35 ^a^	0.31 ± 0.08 ^b^
4	30.2	8-Deoxygartanin	1.40 ± 0.19	tr
5	34.2	Gartanin	2.09 ± 0.67 ^a^	0.04 ± 0.02 ^b^
6	35.1	α-mangostin	58.08 ± 3.86 ^a^	0.86 ± 0.22 ^b^
7	41.6	9-Hydroxycalabaxanthone	1.73 ± 0.41 ^a^	0.43 ± 0.16 ^b^
8	44.0	β-mangostin	0.76 ± 0.34	tr

Values not bearing the same letters across columns are significantly different (*p* < 0.05). * db = dry basis; tr = trace.

**Table 2:** Concentrations of xanthones in the tinctures of mangosteen pericarp and leaves.

Peak No.	Retention Time	Compound	Concentration (mg/g, db *)					
			Pericarp			Leaves		
			80%	60%	40%	80%	60%	40%
1	21.5	Garcinone C	0.43 ± 0.01 ^a^	0.30 ± 0.01 ^b^	0.22 ± 0.01 ^c^	0.04 ± 0.00 ^d^	0.03 ± 0.00 ^e^	0.01 ± 0.00 ^f^
2	24.6	Garcinone D	0.58 ± 0.01 ^a^	0.47 ± 0.01 ^b^	0.27 ± 0.01 ^c^	tr	tr	tr
3	29.2	γ-mangostin	3.02 ± 0.05 ^a^	2.18 ± 0.13 ^b^	0.88 ± 0.09 ^c^	0.26 ± 0.00 ^d^	0.19 ± 0.0 ^e^	0.02 ± 0.00 ^f^
4	30.0	8-Deoxygartanin	0.48 ± 0.01 ^a^	0.44 ± 0.03 ^a^	0.23 ± 0.01 ^b^	0.12 ± 0.01 ^d^	0.10 ± 0.01 ^e^	0.03 ± 0.00 ^f^
5	34.0	Gartanin	0.81 ± 0.02 ^a^	0.60 ± 0.03 ^b^	0.17 ± 0.00 ^c^	0.70 ± 0.01 ^d^	0.41 ± 0.01 ^e^	0.04 ± 0.00 ^f^
6	34.8	α-mangostin	26.33 ± 0.85 ^a^	20.84 ± 1.04 ^b^	1.60 ± 0.16 ^c^	0.98 ± 0.09 ^d^	0.69 ± 0.02 ^e^	0.13 ± 0.01 ^f^
7	41.4	9-Hydroxycalabaxanthone	0.62 ± 0.01 ^a^	0.43 ± 0.02 ^b^	0.11 ± 0.00 ^c^	0.43 ± 0.01 ^b^	0.19 ± 0.01 ^d^	0.02 ± 0.00 ^e^
8	43.8	β-mangostin	0.32 ± 0.01 ^a^	0.46 ± 0.08 ^a^	0.02 ± 0.00 ^c^	tr	tr	tr

Values not bearing the same letters across columns (only for pericarp) are significantly different (*p* < 0.05). * db = dry basis; tr = trace.

## Data Availability

The data presented in this study are available upon request from the corresponding author.

## Abbreviations

The following abbreviations are used in this manuscript:

9-OHCal	9-hydroxycalabaxanthone
ARE	Antioxidant Response Element
BCA	Bicinchoninic Acid
COX-2	Cyclooxygenase-2
CQ	Concentration to Quadruple
D0	Day 0
DAPI	4′,6-diamidino-2-phenylindole
DMEM	Dulbecco’s Modified Eagle Medium
DMSO	Dimethyl Sulfoxide
DSS	Dextran Sulfate Sodium
EDTA	Ethylenediaminetetraacetic Acid
EtOH	Ethanol
FBS	Fetal Bovine Serum
GarC	Garcinone C
GarD	Garcinone D
GSTP1	Glutathione S-transferase Pi 1
HO-1	Heme Oxygenase 1
HPLC	High Performance Liquid Chromatography
HRP	Horseradish Peroxidase
IC50	Half-Maximal Inhibitory Concentration
iNOS	Inducible Nitric Oxide Synthase
LPS	Lipopolysaccharide
MAPK	Mitogen-Activated Protein Kinase
MeOH	Methanol
MTT	3-(4,5-Dimethylthiazol-2-yl)-2,5-diphenyltetrazolium bromide
NAD(P)H	Nicotinamide Adenine Dinucleotide (Phosphate), Reduced Form
NO	Nitric Oxide
NQO1	NAD(P)H Quinone Dehydrogenase 1
Nrf2	Nuclear Factor Erythroid 2 Related Factor 2
PBS	Phosphate Buffered Saline
PLE	Pressurized Liquid Extraction
PVDF	Polyvinylidene Fluoride
ROS	Reactive Oxygen Species
RT	Room Temperature
SDS-PAGE	Sodium Dodecyl Sulfate Polyacrylamide Gel Electrophoresis
SFE	Supercritical Fluid Extraction
UPLC	Ultra-Performance Liquid Chromatography
W9	Week 9
W10	Week 10
W12	Week 12
